# Concomitant Botulinum Toxin Injections for Neurogenic Detrusor Overactivity and Spasticity—A Retrospective Analysis of Practice and Safety

**DOI:** 10.3390/toxins16060252

**Published:** 2024-05-28

**Authors:** Arnaud Leilaz, Charles Joussain, Pierre Denys, Djamel Bensmail, Jonathan Levy

**Affiliations:** 1 Spinal Unit, Department of Physical and Rehabilitation Medicine, Raymond Poincaré Teaching Hospital, APHP Paris Saclay, 92380 Garches, France; arnaud.leilaz@aphp.fr (A.L.); djamel.bensmail@aphp.fr (D.B.); 2 School of Medicine, Sorbonne University, 75013 Paris, France; 3 Neurourology Unit, Department of Physical and Rehabilitation Medicine, Raymond Poincaré Teaching Hospital, APHP Paris Saclay, 92380 Garches, France; charles.joussain@aphp.fr (C.J.); pierre.denys@aphp.fr (P.D.); 4 INSERM 1179, University of Versailles Saint-Quentin-en-Yvcelines, 78180 Montigny-le-Bretonneux, France

**Keywords:** botulinum toxin, spinal cord injuries, multiple sclerosis, spasticity, neurogenic detrusor overactivity, health data hub

## Abstract

As multiple indications for botulinum toxin injections (BTIs) can coexist for neurological patients, there are to date no description of concomitant injections (CIs) to treat both spasticity and neurogenic detrusor overactivity incontinence (NDOI) in patients with spinal cord injuries (SCIs) and multiple sclerosis (MS). We therefore identified patients followed at our institution by health data hub digging, using a specific procedure coding system in use in France, who have been treated at least once with detrusor and skeletal muscle BTIs within the same 1-month period, over the past 5 years (2017–2021). We analyzed 72 patients representing 319 CIs. Fifty (69%) were male, and the patients were mostly SCI (76%) and MS (18%) patients and were treated by a mean number of CIs of 4.4 ± 3.6 [1–14]. The mean cumulative dose was 442.1 ± 98.8 U, and 95% of CIs were performed within a 72 h timeframe. Among all CIs, five patients had symptoms evocative of distant spread but only one had a confirmed pathological jitter in single-fiber EMG. Eleven discontinued CIs for surgical alternatives: enterocystoplasty (five), tenotomy (three), intrathecal baclofen (two) and neurotomy (one). Concomitant BTIs for treating both spasticity and NDOI at the same time appeared safe when performed within a short delay and in compliance with actual knowledge for maximum doses.

## 1. Introduction

Botulinum toxin type A (BoNT-A) is a neurotoxin, naturally produced by *Clostridium botulinum*, which inhibits the release of acetylcholine vesicles into the synaptic cleft of neuromuscular junctions. It has been used for therapeutic purposes since the late 1970s to correct strabismus in infants. Its indications have considerably expanded since then. Among them, spasticity and neurogenic detrusor overactivity incontinence (NDOI) are approved indications worldwide [1,2,3,4,5].

In persons living with spinal cord injuries (SCIs), recent results from the InSCI surveys revealed that more than 75% of respondents reported experiencing spasticity [6], which was considered troublesome by 48.7% [7] and associated with poor quality of life in 46% [8]. In multiple sclerosis (MS), recent findings from the analysis of the French national health data hub, based on ICD-10 codes, revealed that 23.6% of persons suffering from MS were identified as spastic [9]. Surveys based on symptom questionnaires showed up to 82% of patients reporting spasticity. Furthermore, spasticity was inversely correlated with satisfaction regarding life situation and physical health [10].

Similarly, results from InSCI surveys showed that bladder dysfunction was reported by more than 60% of persons with SCI [6], which was associated with poor quality of life by 46.8% [8]. Fifty to eighty percent of persons living with SCI may experience NDOI [11]. In MS, 87% of patients who responded to Hemmett’s questionnaires reported bladder dysfunction [12], and estimations of its prevalence vary widely according to the duration and severity of the disease, from 50% to 90%, with almost all patients with MS of more than 10 years of duration experiencing bladder dysfunction symptoms [13]. In the aforementioned study by Gustavsen, bowel and bladder dysfunction were inversely correlated with life satisfaction, mental health and physical health [10].

In persons living with SCI or MS, both spasticity and NDOI can be efficiently treated with BoNT-A injections (BTIs) [3,5]; hence, both secondary conditions are indications for the same treatment that may coexist in these patients. Given the variation in duration of action between those two indications (3 to 6 months in spasticity and over 6 months in NDOI) [14,15,16,17] and considering the recommended interval before reinjection (3 months for all toxins [1,2,3]) and the immunogenicity risk of early reinjections, a practical approach would involve performing BTIs concomitantly for both indications every 6 months. Injections in striated muscles could be potentially performed 3 months after those CIs and 3 months before the next round. To date, there is no published evidence regarding the concomitant use of BoNT-A to treat spasticity and NDOI in persons with SCI or MS, leaving the following questions unanswered: Is it possible to use different formulations of BoNT-A at the same time? Are maximum doses for each indication cumulative? Do both indications have to be treated on the same day, or within what time interval? Consequently, there are no guidelines or recommendations that specify dose regimens and time intervals between both injection sites and between two concomitant sessions of injections. We aimed to describe the practice of concomitant BTIs for NDOI and spasticity in a referral center for the management of patients with SCI and MS.

## 2. Results

**Figure 1:**
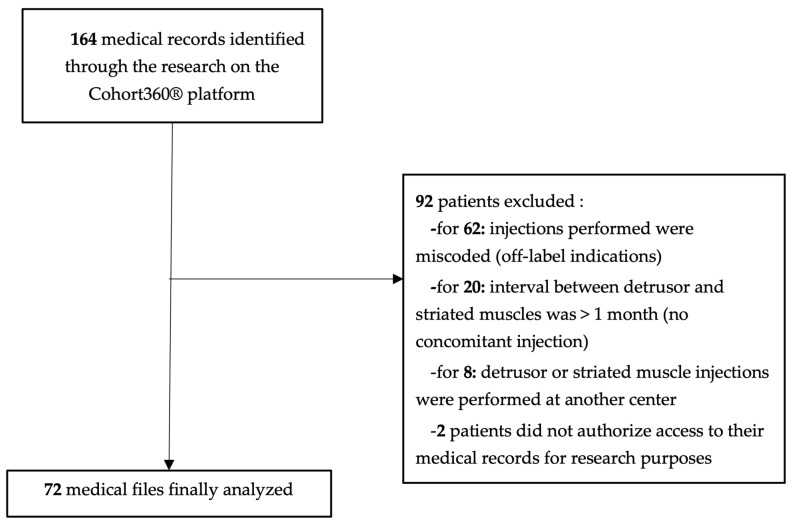
Flow chart for study participants.

We identified 164 patients for whom the codes of interest were recorded over the period 1 January 2017 to 31 December 2021. Among them, 62 were excluded because the procedures performed were miscoded for off-label use of BoNT-A (JDLE332/JDLE900 BTI performed in the urethral sphincter or erectile bodies). For 20 patients, the delay between the detrusor and striated muscle injections was greater than 1 month and did not meet the proposed definition of a concomitant injection (CI). However, we analyzed and described this subpopulation separately. Finally, eight other patients were excluded because some or all of the injections were performed at another center, which prevented us from retrieving data of interest, and two patients objected to the use of their health data. Finally, we included 72 patients whose data were retained for the main analysis (Figure 1).

### 2.1. Patients’ Characteristics

Of the 72 patients included, 22 (31%) were female, and the mean age at first CI was 48.4 ± 13.9 years. There were 56 (78%) patients with SCI, of whom 44 (79%) were paraplegics (Table 1). Most of them had incomplete injuries (*n* = 40, 71% of SCI). For one patient with spina bifida, the precise neurological level of injury (defined with ASIA score) was not specified. Based on the information available, we considered this patient to have incomplete paraplegia. Thirteen patients suffered from MS, with a median EDSS score of 7.5 [6.5; 7.5]. Three patients included (4%) presented with another etiology of the central nervous system lesions (neurosarcoidosis, traumatic brain injury, meningioma).

Oral treatments for spasticity or NDOI associated with BoNT were Baclofen for 29 (40.3%) patients or antimuscarinics for 48 (67%) patients, either as monotherapy (n = 24) or dual therapy (n = 24) (Table 2).

### 2.2. Description of Concomitant Injections

**Figure 2:**
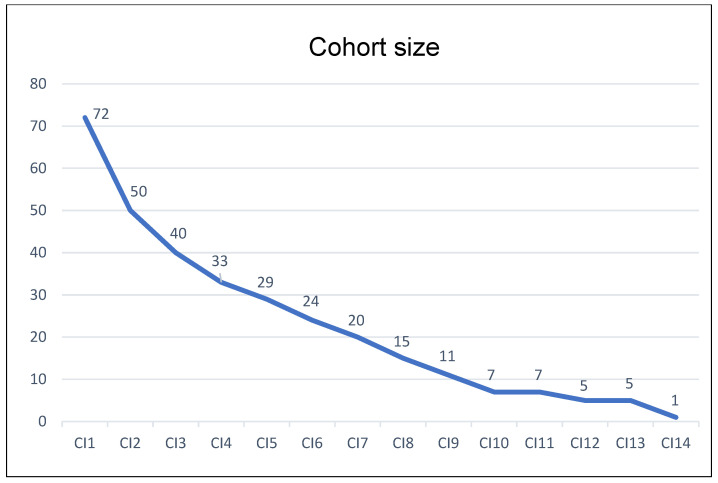
Evolution of cohort size with the number of concomitant injections (CIn: nth concomitant injection).

**Figure 3:**
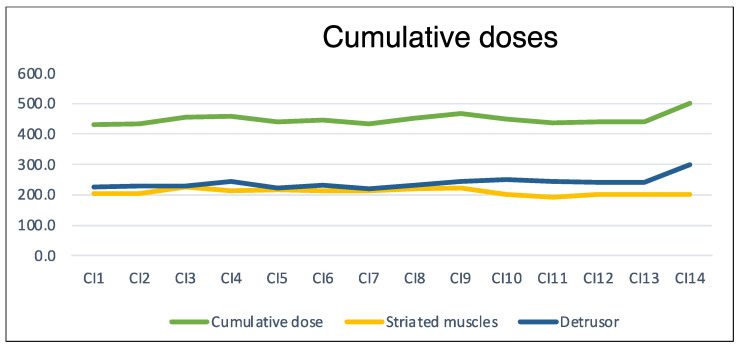
Average doses of botulinum toxin (in Allergan units) during concomitant injections (CIn: nth concomitant injection).

The average number of CIs performed per patient was 4.4 ± 3.6, and the range was (1–14) (Figure 2), representing a total number of 319 CIs. The average time between the onset of pathology and the first CI was 16.2 ± 12.5 years. The mean cumulative dose for all patients over the total number of CIs was 442 ± 99 U. The latter comprised a mean detrusor dose of 231 ± 62 U and a mean striated muscle dose of 211 ± 97 U. The average share of detrusor injections represented 52.2% of the average cumulative dose. The minimum and maximum cumulative doses were 100 and 1000, respectively, and occurred only once each. The median was 450 U with 50% of all CIs being between 400 and 500 U. Twenty-five CIs were performed at 600 U, and nine exceeded 600 U. The doses used were relatively stable over time (Figure 3)

**Figure 4:**
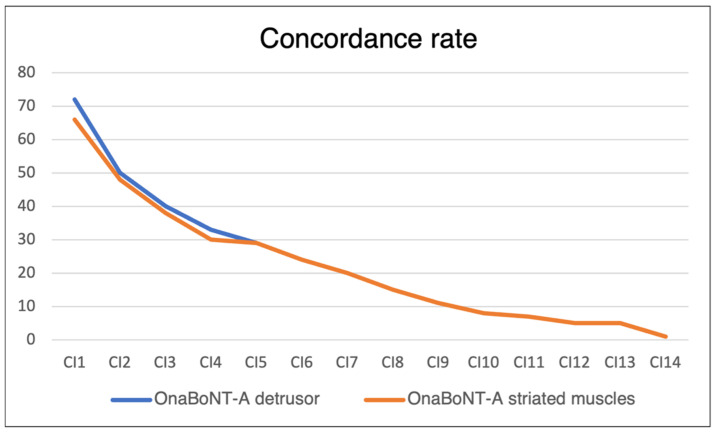
Concordance between BoNT-A types used in the detrusor and striated muscles during CI (CIn: nth concomitant injection).

**Figure 5:**
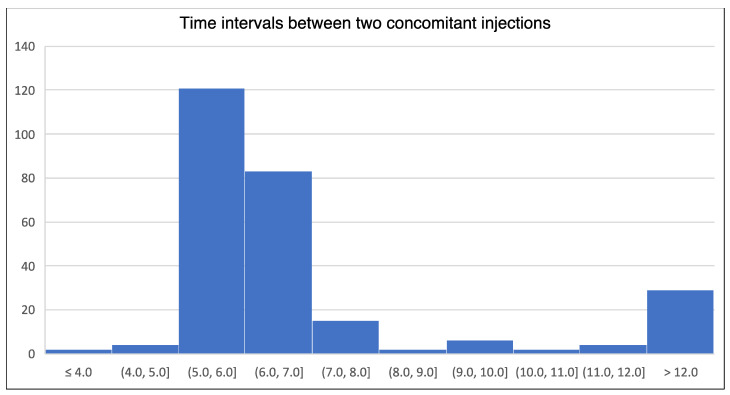
Distribution of time intervals between two concomitant injections.

The median time between the two injection sessions (detrusor and striated muscles) making up the CI was 1 day [1-1], with 95% of CIs being performed within 3 days and 97% within the same week. During CIs, 100% of detrusor injections were performed with OnaBoNT-A, compared with 96.0% of striated muscle injections (4% IncoBoNT-A, 0% AboBoNT-A). The concordance rate ranged from 0.91 to 0.96 for the first four CIs and became equal to 1 afterwards (see Figure 4). The mean time between two CIs was 7.87 ± 5.5 months (Figure 5).

### 2.3. Univariate Comparison between MS and SCI Subgroups

We observed a shorter mean time between diagnosis of the disease and the first concomitant injection in the SCI subgroup compared to MS (14.6 vs. 21.4 years; *p* < 0.01). Patients with SCI received a lower mean dose in striated muscles (202 vs. 267 U; *p* < 0.05) and were younger (46.5 vs. 56.2; *p* < 0.05) than patients with MS at first CI. There was no difference between the two groups in terms of the mean number of concomitant injections, total cumulative dose or detrusor dose. These results are summarized in Table 3.

### 2.4. Intersessions of Intramuscular BTI

The mean dose of BoNT-A used during intersessions (ISs) was 275 ± 133 U, tending to be higher and more variable than the mean dose used in striated muscles during CIs. The type of BoNT used was OnaBoNT-A in 92% of cases, the remaining 8% being IncoBoNT-A. The average duration between two intersessions was 7.2 ± 2.9 months, while the average interval between IC and IS was 3.2 ± 0.7 months.

### 2.5. Safety and Evolution

We observed five (6.9% of included patients, 1.6% of total number of CIs) suspected cases of pseudobotulism. One (1.4% of included patients, 0.3% of total number of CIs) was confirmed by an electroneuromyogram (ENMG) with altered neuromuscular jitter in the *orbicularis oculii* muscle and led to the discontinuation of BTIs. Another strong suspicion of pseudobotulism led to discontinuation of BTIs, without ENMG exploration. The other three patients proceeded with CIs without complication, as clinical history, timing and ENMG ruled out the hypothesis of a distant spread of BoNT-A. In the remaining 67 (93%) patients and 314 (98%) CIs, no adverse event related to botulinum toxin use was reported. Of note, none of those five cases of suspicion of pseudobotulism exceeded a cumulative dose of 500 U.

Forty patients (56% of the initial study population) discontinued CIs before the data collection deadline (31 December 2021). For 23 patients (32% of the initial population), discontinuation of striated muscle injections was the reason for discontinuation of CIs. The two most common reasons for discontinuation were reconsideration of the initial indication (n = 11, 15.2%) and insufficient therapeutic benefit (n = 6, 8.3%). Four patients (5.5%) underwent neuro-orthopedic surgery, and two (3.3%) had an intrathecal baclofen pump implantation.

Four (5.5%) patients stopped detrusor BTIs and underwent bladder augmentation surgery. The second and third reasons for discontinuation were bladder cancer (n = 2; 2.8%) and reconsideration of the initial indication (n = 2; 2.8%).

Finally, for nine (12.5%) patients, discontinuation of CIs was not specific to either indication, including four (6.9%) patients lost to follow-up.

## 3. Discussion

In this study, we described for the first time the practice of concomitant BoNT-A injections for two main indications in persons living with SCI or MS. This practice appeared to be safe even at high cumulative doses (above 600 U of Botox equivalent) for both indications and within a 72 h timeframe, with only five suspicions (and one confirmed) of distant spread among 321 cycles of concomitant injections.

### 3.1. Maximum Dose per Session

There is currently no consensual recommendation for the maximum dose in CIs, and various approaches can be considered. One approach (let us call it liberal) would be to sum up the maximum recommended doses for each indication, hence 600 U of OnaBoNT-A (400 U for spasticity and 200 U for NDOI) or 2300 US for AboBoNT-A (1500 US for spasticity and 800 US for NDOI) if we stick to drug approvals [2,3] and 900 U for OnaBoNT-A when considering professional consensus [18,19,20] ensuring a safe use. Another approach (we could call it restrictive) would be to use the higher recommended dose between both indications and allocate it to the striated muscles and the detrusor, in line with the latter maximum published dose (300 U of OnaBoNT-A or 800 of AboBoNT-A).

At our institution, we decided to limit doses for concomitant injections at 600 U, which is illustrated by the mean cumulative dose of 442 ± 98.8 U with a detrusor dose rate slightly above 50%. Of note, at our institution, NDOI is usually treated by injections of 200 U of Botox in compliance with drug approval, up to 300 after multidisciplinary discussion, and at the discretion of the injector for the treatment of spasticity.

During intersessions, the mean dose injected within striated muscles for spasticity tended to be higher and more variable than during CIs for the same indication, suggesting that practitioners adjusted their injection strategies for striated muscles during CIs by focusing on specific key muscles while adopting a more global approach during intersession injections.

### 3.2. Time between Injections

We did not find any evidence in the literature regarding an optimal time frame for concomitant injection of BoNT for two indications whether in humans or animals.

The short 72 h period is proposed to limit the development of neutralizing antibodies (NAbs). In 2019, Mathevon et al. published a systematic review of BoNT-induced immunogenicity in spasticity, confirming that a short delay before reinjection (<3 months) was one of the risk factors for NAb production, alongside the dose per injection and the duration of treatment [21]. Although the literature is discordant about the clinical role of NAbs, we applied the precautionary principle with regard to the interval between the two injections making up the concomitant injection. We noted a relative respect for this rule, with there being only 15 (4.7%) CIs out of the 319 analyzed for which the delay between the two sessions was greater than 72 h. No adverse effect was reported for the 12 patients who had one or more CIs with a delay > 72 h between the two injection sessions, and they did not become secondary non-responders. Among the 23 patients who discontinued striated muscles BTI, 12 of them had a decrease in BTI benefit, underwent neuro-orthopedic surgery or had a baclofen pump implantation, suggesting a possible secondary failure. However, it remained difficult to be precise enough in a retrospective analysis.

### 3.3. Time between Two Injection Sessions

For all three BoNT formulations, the main drug approval administrations worldwide have set the minimum time between two injections at 12 weeks [1,2,3,4,5]. In a meta-analysis published in 2022, Ojardias et al. showed that in post-stroke spastic hypertonia, peak efficacy ranged between 4 and 5 weeks following BTI, depending on the formulation used. The half-life varied according to the preparation but was consistent with a reinjection once every 12 weeks [16]. It should be noted that this 12-week time interval before reinjection could not always correspond to that desired by patients with spasticity. In 2014, Bensmail et al. showed that 43.4% of patients included in their study and treated for post-stroke spastic hypertonia would have preferred a reinjection interval of less than or equal to 10 weeks [22].

Regarding NDOI, Rovner et al. demonstrated in 2016 that the median duration of efficacy for OnaBoNT-A injections in the detrusor was approximately 9 months, with 26% of patients experiencing therapeutic effectiveness lasting beyond 12 months [17]. Joussain’s cohort showed shorter mean delays than Rovner’s between two detrusor BTIs from 5.1 ± 1.4 months for 200 U to 6.5 ± 1 months for 300 U. In our study, the average time between two CIs was 7.87 ± 5.5 months, and the average time between a concomitant injection and an intersession was 3.2 ± 0.7 months.

### 3.4. Types of BoNT Used

A pragmatic approach would favor the use of the same drug to treat both indications simultaneously in the same patient. This is in line with the restrictive strategy for cumulative doses. However, one could consider using two different toxins for each indication from a more liberal point of view considering that each injection site could be considered separately and treated with the more appropriate toxin. To date, there is no published evidence to support or reject this strategy.

Nonetheless, it would be valuable to conduct a more comprehensive assessment of the simultaneous administration of IncoBoNT-A in striated muscles alongside OnaBoNT-A or AboBoNT-A in the detrusor, as this approach theoretically carries a risk profile similar to that of exclusive AboBoNT-A or OnaBoNT-A use.

In our center, the concordance rate between the formulations used ranged from 90.9% to 96% for the first four CIs, rising to 100% by the fifth one and afterward. The use of OnaBoNT-A in the detrusor associated with IncoBonT-A in striated muscles was the only discrepancy encountered in our study without reported adverse events.

### 3.5. Concomitant Injections for Other Indications

Other indications (either validated or approved or not) could have been considered in our population of interest, such as neuropathic pain, erectile dysfunction and sialorrhea [23,24,25,26]. However, we focused our analysis on both spasticity and NDOI, which are frequent and well-validated, with known efficacy and safety profiles in our population of interest. Our study was deemed to answer an almost daily interrogation for those who take care of patients with SCI or MS. To our opinion, our study has two advantages. First, as a preliminary descriptive study, it sets a basis for future prospective and interventional studies. Second, it proposes a relatively safe framework for the management of the simultaneous treatment of multiple indications for BTIs, even though it suffers from several limitations.

### 3.6. Study Limitations

We identified patients from our institution’s health data hub using a medical procedure coding system, a method that is nearly comprehensive and systematically conducted by practitioners. This approach offered more robust screening compared to manual methods within our care setting. It is important to note that the coding system, primarily used for medico-economical purposes, may vary among practitioners who may be more or less sensitive to its interest, as already discussed elsewhere [9]. However, off-label use of BoNT-A may not match with a specific code, and for 38% of our initial cohort, JDLE codes were not used for bladder injections but for other pelvic–perineal sites of injections (urethral sphincter and corpora cavernosa). The secondary textual search and analysis of medical files allowed us to exclude the latter and precisely define our population of interest.

Second, this was a single-center study, introducing a recruitment bias. We aimed to observe real-world applications of CIs. We are well aware that only a few highly specialized centers in France perform both striated muscle and detrusor BTIs. Hence, our results may not be applicable anywhere else.

We did not propose a longitudinal approach, which prevented us from conducting a similar analysis to Joussain et al. for treatment discontinuation and withdrawal over time [27].

Finally, the data analysis period partially overlapped with the SARS-CoV-2 pandemic period. This period led to the reorganization of the Physical Medicine and Rehabilitation departments and altered procedure prioritization. We did not explicitly analyze data related to the cancellation and rescheduling of injection sessions. However, a noticeable trend emerged, with a preference for detrusor BoNT injections over striated muscle injections, which were more frequently canceled during this period. We may have underestimated the number of multiple concomitant indications of BoNT-A. Of note, no subjects discontinued CIs and especially intramuscular BTIs without an alternative treatment, and all patients remained followed-up.

## 4. Conclusions

This was the first description of the concomitant use of BoNT-A for both spasticity and NDOI in a population of persons living with SCI or MS, in a referral tertiary center. We proposed a practical framework for multiple-site injections, considering a restrictive strategy for doses, delays and type of toxin, and coordinated CIs for spasticity and NDOI which appeared to be safe. A national survey could help understand how other specialized centers manage these concomitant injections and define guidelines based on professional agreements that could set a basis for interventional studies.

## 5. Materials and Methods

### 5.1. Study Design and Location

We conducted a retrospective, monocentric cohort study observing practices of concomitant BoNT injections in MS and SCI patients with an inclusion period spanning from 2017 to 2021 and data collection from 2012 to 2021.

This study was carried out in a referral center for the management and follow-up of patients with MS and SCI.

### 5.2. Population

To identify eligible patients, we used the Cohort360^®^ healthcare platform (Assistance Publique des Hôpitaux de Paris). This platform, linked to our institution’s health data hub, enabled us to define a cohort of patients based on demographic and nosological criteria and coded medical procedures. Coding is carried out in accordance with the French version of the 10th International Classification of Diseases (ICD-10-F) and the Joint Classification for Medical Procedures (*Classification Commune Des Actes Médicaux*—CCAM). Any patient registered at least once in our department and met the inclusion criteria defined below could be included in the cohort, with “pseudonymized” access to their medical file and health data.

We arbitrarily defined concomitant injection (CI) as one session of BoNT injection in the detrusor and in the striated muscles, within a one-month period in the same patient. The sole inclusion criterion was the completion of at least one CI. We excluded patients under 18 years of age at the time of the first CI, injection sessions performed in other centers, or miscoded for indications other than the detrusor and striated muscles (e.g., urethral sphincter, erectile bodies, subcutaneous injections). Patients who did not consent to the use of data from their medical records were excluded.

### 5.3. Cohort Identification Method

The first step of our study was to set up the cohort using Cohort360^®^. Eligible patients were identified by searching for CCAM codes corresponding to BoNT-A injections in the detrusor (JDLE332 (JDLE 332: Urethrocystoscopic injection of botulinum toxin into the bladder musculature) or JDLE900 (JDLE 900: Urethrocystoscopic injection of botulinum toxin into the bladder musculature. Code applied from 2005 to 2014)) and striated muscles (PCLB002 (PCLB002: transcutaneous injection of botulinum toxin into striated muscles, without electromyographic detection test) or PCLB003 (PCLB003: transcutaneous injection of botulinum toxin into striated muscles, with electromyographic detection test)). The JDLE 900 code was deleted in 2014 but retained in the search equation to cover any updating shortfalls. The patients screened for inclusion in the cohort were those in the scope of care for whom codes JDLE332/JDLE900 and PCLB002/PCLB003 had been filled in between 1 January 2017 and 31 December 2021. Second, we performed a text search within medical files to look for inclusion and exclusion criteria.

### 5.4. Data Collection

Once patients were included, we reviewed their medical files to collect data of interest over the period spanning from 1 January 2012 to 31 December 2021. We collected data on patient characteristics (gender; date of birth), pathology (date of onset of pathology; ASIA score and etiology for SCI patients; EDSS for MS patients), injection sessions (date; type and dose of BoNT used in striated muscles and detrusor; cumulative dose and time between sessions; total number of CIs). Cumulative dose was defined as the sum of the striated muscle and bladder doses. We referred to ‘Botox equivalent’ as a standardized dose unit system in this study. We also studied injection sessions performed solely in striated muscles between two CIs, which we defined as “intersessions” (ISs) and for which we recorded the dates, doses and types of BoNT used. Associated therapies for spasticity (drug treatment; chemical or mechanical neurolysis, reversible or definitive; surgical treatments for muscle hypo-extensibility and pressure sores) and NDOI (anticholinergic treatments; bladder augmentation surgeries with or without urine diversion) were also reported.

We looked at safety data (adverse events reported; reasons for discontinuation of CI). For any suspected cases of BoNT-A distant spread syndrome, we used a definition proposed by our team defining a functional form and a severe form for putative pseudobotulism [Supplementary Materials]. EDSS data and data regarding associated therapies were collected using the latest information available on 31/12/2021.

### 5.5. Outcome and Judgment Criteria

Our main objective was to describe the practice of concomitant injections in terms of doses used (cumulative, in each indication and during ISs), time intervals (between two sessions, two CIs), number of sessions performed and safety (adverse effects, reasons for treatment discontinuation).

### 5.6. Statistical Analysis

Continuous variables are expressed as means with standard deviations or medians with interquartile ranges (IQRs), ordinal categorical variables are expressed as medians with IQRs, and categorical variables are described as absolute numbers and percentages.

We were interested in concordance with the type of BoNT used in striated muscles during CIs, and we defined the concordance rate as follows: for each CI, this rate was 1 if all patients had received the same type of BoNT in both the bladder and striated muscles, and the minimum CR was 0.5.

We carried out comparative statistics between MS and SCI groups. For this purpose, we used Student’s *t*-test to compare means, after performing an F-test to check the comparability of variances, assuming a normal distribution for all. Categorical variables were compared using the Chi-square test.

BoNT doses were expressed in equivalents of Allergan units (U) for OnaBoNT-A and IncoBoNT-A and in Speywood units (US) for AboBoNT-A. One unit (U) corresponds to the lethal dose 50 (LD50) of the product reconstituted and injected intraperitoneally into mice. For the sake of uniformity, dose units are presented in “Botox equivalent” (U), where IncoBonT-A = OnaBonT-A and AboBoNT-A = 2.5 OnaBoNT-A [28].

### 5.7. Ethical Aspects

The study was conducted in compliance with French bioethics laws. Medical data from each patient’s medical documents were coded and pseudonymized within the health data hub. Our institution’s health data hub has been approved by the French Data Protection Authority under No. 1980120. Patients were informed of the use of their data for scientific research purposes by a specific statement generated on every medical report from our institution, and of the possibility to object to it via written declaration.

## Figures and Tables

**Table 1:** General characteristics of patients.

	n	%
Men	50	69
Women	22	31
SCI	56	78
Paraplegia	44	79
Complete	16	36
Incomplete	28	64
Tetraplegia	12	23
Complete	0	0
Incomplete	12	100
Traumatic causes	36	64
MS	13	18
EDSS < 7.5	5	38
EDSS ≥ 7.5	8	62
Other pathologies	3	4

SCI: spinal cord injury; MS: multiple sclerosis; EDSS: expanded disability status scale.

**Table 2:** Therapeutics associated with botulinum toxin injections.

	n	%
Baclofen per os	29	40.3
Intrathecal baclofen	3	4.2
Chemical neurolysis	7	9.7
Anticholinergics	48	66.7
Monotherapy	24	50
Dual therapy	24	50
Bladder surgery	11	15.3
including		
Bladder augmentation	9	81.8
Mitrofanoff-type continent shunts	2	18.2
Pressure sore surgery	11	15.3
Neuro-orthopedic surgery	12	16.7

**Table 3:** Subgroup analysis of spinal cord injury and MS.

	MS (n = 13)		SCI (n = 56)		*T*-Test
	Mean	SD	Mean	SD	
Age at 1st CI (a)	56.2	9.6	46.5	14.2	*p* < 0.05
Diagnostic delay—1st CI (y)	21.4	6.3	14.6	13.1	*p* < 0.01
Number of CIs (U)	3.8	2.9	4.4	3.8	*p* > 0.05
Cumulative dose (U)	498	119.5	436.5	86.9	*p* > 0.05
Striated muscle dose (U)	267.0	83.7	201.7	95.7	*p* < 0.05
Detrusor dose (U)	231.0	93.0	234.8	50.2	*p* > 0.05

MS: multiple sclerosis; SCI: spinal cord injury; CI: concomitant injection; *T*-test: Student *T*-test.

## Data Availability

Data are available upon reasonable request to the corresponding author.

## Supplementary Materials

The following supporting information can be downloaded at: https://www.mdpi.com/article/10.3390/toxins16060252/s1, Table S1: symptoms of severe and functional (mild) forms of pseudobotulism, as proposed by Karabulut et al.
