# Supplementary material for: Concomitant Botulinum Toxin Injections for Neurogenic Detrusor Overactivity and Spasticity—A Retrospective Analysis of Practice and Safety
Source: Toxins (Basel). 2024 May 28;16(6):252. doi: 10.3390/toxins16060252 (PMC11209118; doi:10.3390/toxins16060252)
Supplement: Supplementary file 1 [file toxins-16-00252-s001.zip › toxins-2940597-supplementary.pdf]

**Supplementary Materials**

| Severe form of pseudobotulism | Functional form of pseudobotulism |
|---|---|
| Respiratory symptoms<br>Ocular symptoms<br>Sane limb weakness<br>ENT symptoms<br>Asthenia | Global weakness causing falls<br>Degredation of transfers<br>Distant neurogenic limb weakness |

*Based on Dr C. Karabulut's work, symptoms identified in patients who underwent single fiber EMG for suspicion of pseudobotulism, and analysed with a principal component analysis. Presented at the 2022 SOFMER Congress.*
